# App-based intervention among adolescents with persistent pain: a pilot feasibility randomized controlled trial

**DOI:** 10.1186/s40814-022-01113-0

**Published:** 2022-07-27

**Authors:** Erik Grasaas, Sølvi Helseth, Liv Fegran, Jennifer Stinson, Milada Småstuen, Chitra Lalloo, Kristin Haraldstad

**Affiliations:** 1 Department of Health and Nursing Science, Faculty of Health and Sport Sciences, University of Agder, Kristiansand, Norway; 2 Department of Nursing and Health Promotion, Faculty of Health Sciences, Oslo Metropolitan University, Oslo, Norway; 3 Child Health Evaluative Sciences, The Hospital for Sick Children, Toronto, Canada; 4 Lawrence S. Bloomberg Faculty of Nursing, University of Toronto, Toronto, Canada; 5 Institute for Health Policy, Management, and Evaluation, University of Toronto, Toronto, Canada

**Keywords:** Chronic pain, Adolescent, Randomized controlled trial, Mobile applications, Self-management

## Abstract

**Background:**

Persistent pain in adolescence adversely affects everyday life and is an important public health problem. The primary aim was to determine the feasibility of an 8-week app-based self-management intervention to reduce pain and improve health-related quality of life in a community-based population of adolescents with persistent pain. A secondary aim was to explore differences in health outcomes between the intervention and control groups.

**Methods:**

A sample of 73 adolescents aged 16–19 years with persistent pain from a community-based population were randomized into 2 groups. The intervention group received the Norwegian culturally adapted version of the *iCanCope with Pain*^TM^ app, which includes symptom tracking, goal setting, self-management strategies, and social support. The attention control group received a symptom tracking app. Feasibility was assessed as attrition rates and level of engagement (interactions with the app). The secondary outcomes included pain intensity, health-related quality of life, self-efficacy, pain self-efficacy, perceived social support from friends, anxiety and depression, and patient global impression. Statistical analyses were conducted using SPSS.

**Results:**

Demographic and baseline outcome variables did not differ between the 2 groups. No differences were found between the participants completing the study and those who withdrew. Twenty-eight adolescents completed the intervention as planned (62% attrition). Both groups had a low level of app engagement. Intention-to-treat analysis (*n* = 19 + 14) showed no significant differences in outcomes between groups. However, the large effect size (Cohen’s *d* = .9) for depression suggested a lower depression score in the intervention group.

**Conclusions:**

High treatment attrition and low engagement indicate the need for changes in trial design in a full-scale randomized controlled trial to improve participant retention.

**Trial registration:**

The iCanCope with Pain Norway trial was retrospectively registered in Clinical Trials.gov (ID: NCT03551977). Registered 6 June 2018.

**Supplementary Information:**

The online version contains supplementary material available at 10.1186/s40814-022-01113-0.

## Key messages regarding feasibility

- This study builds on an earlier cultural adaptation and is the first study to determine the feasibility of a self-management app aiming at reducing pain and improve HRQOL in a school-based population of adolescents with persistent pain in Norway.
- The high treatment attrition and low engagement rates observed indicate the need for some changes in trial design in a full-scale randomized controlled trial to improve participant retention.
- Possible changes in trial design could be including personal support and use of additional electronic platforms for communication with participants. Nevertheless, our findings provide estimates for calculation of sample sizes in future app-based intervention trials of school-based adolescents with persistent pain.

## Background

Persistent pain in adolescents adversely affects everyday life and is an important public health problem [1]. Pain lasting ≥ 3 months is classified as persistent/chronic pain [2] and is prevalent in about 20–35% of adolescents in Western countries [3–7]. The etiology for many forms of persistent pain is often unknown [2]. Understanding persistent pain in adolescence is complexed and thus often viewed through a biopsychosocial model, as pain is influenced by several interacting factors [8]. There are several potential underlying mechanisms and mediators associated with persistent pain in adolescence, such as physical, psychological, social, and economic relations [1, 9, 10]. Pain is often referred to as a vicious circle [11] in this age group because it negatively amplifies other aspects of life, which again influences the pain experiences. Previous studies have reported impaired health-related quality of life (HRQOL) in adolescents with persistent pain compared with those without pain [12, 13]. Adolescents in pain often do not know where to seek information or how to cope with pain [14, 15]. The Internet has become a source of advice and provides self-management skills for younger people to cope with pain [14].

Systematic reviews indicate that Internet-delivered self-management interventions can help reduce pain and/or improve HRQOL in children and adolescents with persistent pain [16, 17]. However, most adolescents in these studies were recruited from a clinical setting, where the adolescents already are undergoing treatment. Because some adolescents experience barriers when accessing traditional therapies, such as talking in groups or with health personnel [18], some might prefer remotely delivered interventions for coping with pain.

The use of smartphone applications (apps) may be preferred for receiving digital health information, and hundreds of pain management apps are available for the public [19]. A systematic review of the benefits of apps for pain management in different age and patient groups concluded that pain management apps may be beneficial for patients, particularly in community-based settings [20]. However, most pain apps available for the public have not been evaluated scientifically [21]. There is a need for research on evidence- and theory-based app interventions aimed at reducing pain and increasing HRQOL among a school-based population of adolescents with persistent pain.

Researchers in collaboration with eHealth Innovation at University Health Network (Toronto, Canada) developed an evidence- and theory-based pain self-management app for adolescents called *iCanCope with Pain*^*TM*^ [18]. We have translated and culturally adapted the English-language *iCanCope with Pain*^*TM*^ app into the Norwegian context and evaluated the app’s usability in laboratory settings and in the field [22]. The previous usability testing of the Norwegian version of the iCanCope with Pain^TM^ app was limited to 2 weeks of use and included 5 participants. We found that end users in the previous usability in the field test reported high system usability score with only minor errors [22]. These findings suggested that few changes were needed in the app before a pilot trial could proceed.

The purpose of this study was to determine the feasibility of the Norwegian *iCanCope with Pain*^*TM*^ app in an 8-week pilot randomized controlled trial (RCT) aimed at reducing pain and improving HRQOL in a school-based population of adolescents with persistent pain. The primary aim was to determine the trial feasibility in terms of participant attrition rates and level of engagement with the app. The secondary aim was to determine the impact of the intervention on pain intensity, HRQOL, self-efficacy, pain self-efficacy, perceived social support from friends, anxiety and depression, and patient global impression. We hypothesized that adolescents receiving a mobile phone app containing strategies for coping better with pain would experience improvements in outcomes compared with adolescents receiving an attention control app.

## Methods

### Design

This present study used a pilot RCT design with 2 parallel groups. The intervention group received the *iCanCope with Pain*^*TM*^ app comprising 4 evidence- and theory-based features: (I) symptom trackers for pain intensity, pain interference, sleep, mood, physical activity, and energy; (II) goal setting to improve pain and function; (III) a toolbox of pain education and self-management strategies; and (IV) peer-based social support via a monitored community forum. The attention control group received an app that included symptom trackers only. Both groups were asked to use the respective apps for 8 weeks. Outcome measures were examined at the baseline (T0) and after the intervention (T1).

### Participants

The study was conducted in Southern Norway in 2018. Five government-funded high schools were invited to participate, and all agreed. Eligible individuals were adolescents aged 16–19 years with self-reported persistent pain, defined as weekly pain lasting 3 months or longer. They were able to read and understand Norwegian and had their own smartphone (iPhone or Android). Adolescents with cognitive disability were excluded because of their inability to understand how to use the *iCanCope with Pain*^*TM*^ app independently. Adolescents with a diagnosis of a pathological or medical origin (e.g., oncology patients) were excluded because the program was not designed specifically for these pain conditions.

### Procedure

The first author visited the 5 high schools and explained the study in each classroom. To ensure anonymity and confidentiality, adolescents received a written brochure with an attached email address generated for this purpose only. Information was also available on the websites of each participating high school. Both the oral presentation in the classroom and written information included the inclusion and exclusion criteria of the study. Any adolescents who experienced persistent pain and wanted to participate in the study were instructed to send an email to the corresponding study email address. When the baseline questionnaire was completed, the randomization took place and the last author sent the eligible participants their corresponding username, password, and a short PowerPoint presentation about downloading and using the app. The first author was blinded to the simple randomization procedure performed by the last author using a computer-generated randomization list and the generated coding sheets. Herein, identification number of the participant and method of treatment was randomly selected and placed in numbered envelopes, which was identical and opaque for both groups. After the 8-week intervention period, a link to the online postintervention questionnaire was sent to each participant’s email address.

### Outcomes

The primary outcome of this study was the feasibility of using the Norwegian *iCanCope with Pain*^*TM*^ app in a school-based population of adolescents with persistent pain measured. Specific feasibility outcomes were:

1. Attrition rate was defined as the percentage of participants who failed to complete the final measures.
2. App engagement was measured as the total number of completed symptom check-ins (feature I) over the study period.

The secondary study outcomes, focused on preliminary effectiveness, were:

*Pain* was assessed using the Lübeck Pain-Screening Questionnaire (LPQ), which has, as a measure of internal consistency, a Cronbach’s alpha of 0.92 [3]. The LPQ includes a visual analog scale (VAS) for participants to assess their pain intensity at the present moment, and the score ranged from 0 (no pain) to 10 (worst pain imaginable) [3]. The VAS slider is often used as a measure of pain intensity and has been found to be both valid and reliable, including its digital use [23].

*HRQOL* was measured using KIDSCREEN-52, which is a cross-cultural multidimensional instrument that has been validated in several countries; the internal consistency, as measured by Cronbach’s alpha, is > 0.80 for all dimensions [24–26]. The questionnaire includes 52 items grouped into 10 subscales, which are scored using a 1–5 Likert scale. The electronic format of the survey ensured that all items required answers, which resulted in no missing data. We followed the KIDSCREEN manual and transferred negative questions into positive [24]. The data were then transformed linearly to a 0–100-point scale, where 0 indicated the lowest and 100 indicated the highest HRQOL.

*Self-efficacy* was measured using the General Perceived Self-Efficacy Scale short form (GSE) [27]. The short form has been found to be both valid and reliable and has a satisfactory internal consistency of 0.82 [28]. The GSE is often expressed as the global confidence in one’s ability across a wide range of demanding and novel situations [29]. All items use a 1–4-point scale, where 1 refers to the lowest GSE and 4 the highest, giving a total score from 5 to 20.

*Anxiety and depression* were measured using the Hospital Anxiety and Depression Scale Questionnaire (HADS) [30]. The HADS is a validated method for assessing the symptom severity of anxiety disorders and depression, and it has a satisfactory internal consistency of 0.77–0.89 [31]. The HADS total score (HADS-T) is based on 14 items separated into 2 subscales: anxiety and depression (HADS-D). Each subscale comprises 7 items that are rated on a Likert scale of 0–3. The total for each subscale is 0–21, and the total HADS score is 0–42. Lower values reflect less anxiety and depression.

The following three instruments were translated into Norwegian based on the principles of good practice for translation and cultural adaptation explained by Wild et al. [32].

*Perceived social support* was measured using the Perceived Social Support–Friends Scale (PSS-Fr) questionnaire to measure adolescents’ social support levels [33]. The internal consistency was 0.84 (Cronbach’s alpha). The PSS-Fr has been shown to be a valid and reliable instrument among adolescents. It includes 20 statements that refer to feelings and experiences that occur to most people at one time or another in their relationships with friends [33]. There are 3 answers for each statement: yes, no, and do not know. These measures were categorized numerically as yes = 1 and no and do not know = 0. They yielded a total scale of 0–20, where higher values represent greater perceived social support.

*Pain self-efficacy* was measured using the Pain Self-Efficacy Questionnaire (PSEQ) to assess how confidently the adolescents performed a range of activities described despite their pain [34]. The internal consistency was 0.93 (Cronbach’s alpha). The PSEQ includes 10 items rated on a 7-point Likert scale, where 0 = not at all confident and 6 = completely confident, which gives a total score of 0–60. The PSEQ has been shown to have satisfying psychometric properties [34].

*Global impression of change* was measured using the Patients’ Global Impression of Change Scale (PGIC), which asks participants to self-assess their change in symptoms after the intervention. The PGIC includes 1 question (internal consistency not applicable) and is a validated scale for interpreting the subjective outcome measure of an intervention [35].

### Ethics

The study was approved by the Norwegian Regional Committee for Medical Research Ethics South-East-B (REK reference 2017/350). All participants received oral and written information and signed an informed consent before participating in the study. They were aware that they could withdraw at any time during the study without a reason and that confidentiality and anonymity of their data were ensured at all times. The adolescents did not receive any compensation for participation.

### Data analysis

All statistical analyses were conducted using SPSS version 25 for Windows (IBM Corp), except the computer-generated randomization list and coding sheets, which was conducted using STATA software version 16 (StataCorp). Baseline categorical data (T0) are presented as frequencies and percentages. Continuous data are presented as mean, standard deviation (SD), and effect size (Cohen’s *d*).

Rates of attrition were calculated as participants completed the post-questionnaire (T1) divided by the number of participants at T0. Completion of daily symptom check-ins was used as a proxy for app engagement as this was the only comparable feature received for both the attention control group and the intervention group. Operational definitions were used to categorize check-in engagement over the 8-week (55-day) study period: low engagement, ≤ 24% (≤ 13/55 reports); low-moderate engagement, 25–49% (14 to 27/55 reports); high-moderate engagement, 50–75% (28 to 41/55 reports); and high engagement, 76–100% (42 to 55/55 reports) [36].

All app interactions were measured separately for each feature and are presented as the median and range and as frequencies and percentages for categorical data. All participants were included in the final analysis following the intention-to-treat (ITT) protocol. A general linear model was fitted to explore possible differences in outcomes between the groups. In this model, the post intervention measures were entered as the dependent variables, and these were compared between the treatment groups using the T0 score as the covariate. Effect sizes were determined and are expressed as Cohen’s d as follows: 0.2, small effect; 0.5, medium effect; and 0.8, large effect [37]. *p* values < .05 were considered to be significant.

## Results

### Sample characteristics

Participants at T0 included 73 adolescents aged 16 to 19 years with persistent pain. Most participants were female with multisite pain, in an average of 4.4 pain sites. Almost half of the participants reported pain lasting more than 12 months (47%), about one-third reported having pain daily (27%), and almost half experienced pain several times a week (47%). The characteristics of the sample, both overall and by groups, including pain location, at T0 are presented in Table 1.

**Table 1:** Characteristics of the sample (*N* = 73) at T0

Demographic characteristics	Total *N* = 73 *N* (%)	Intervention *N* = 41 *N* (%)	Control *N* = 32 *N* (%)
Sex (female)	60 (82.2)	36 (87.8)	24 (75.0)
Age	17.4 (1.0)	17.5 (1.0)	17.3 (1.0)
*Pain location*			
Head	64 (87.7)	37 (90.2)	27 (84.4)
Teeth	14 (19.2)	7 (17.1)	7 (21.9)
Ears	13 (17.8)	10 (24.4)	3 (9.3)
Throat	33 (45.2)	21 (51.2)	12 (37.5)
Back	45 (61.6)	25 (61.0)	20 (62.5)
Chest	20 (27.4)	13 (31.7)	7 (21.9)
Abdomen	47 (64.4)	29 (70.7)	18 (56.3)
Reproductive tract	48 (65.8)	28 (68.3)	20 (62.5)
Arms	10 (13.7)	5 (12.2)	5 (15.6)
Legs	27 (37.0)	13 (31.7)	14 (43.8)

### Feasibility analyses

**Fig. 1:**
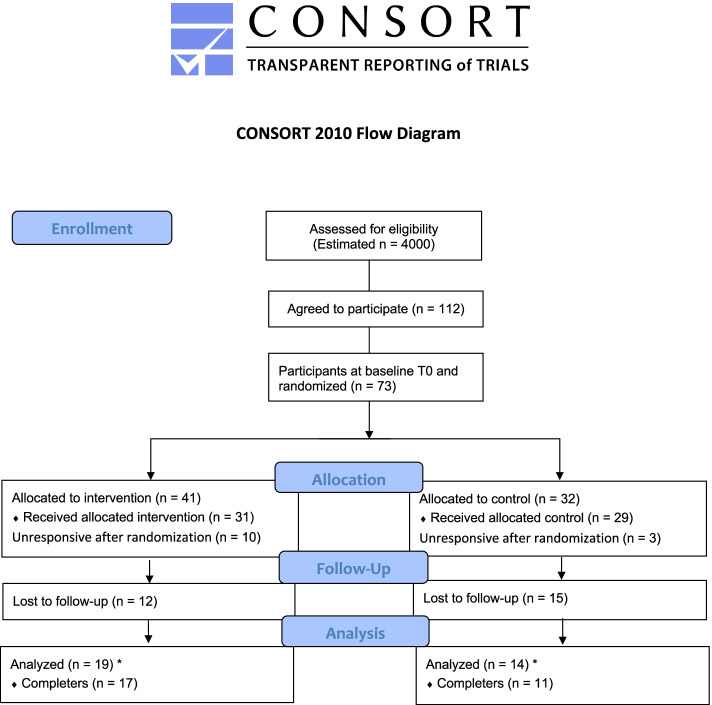
CONSORT flow diagram. *Completers answered all items in the postintervention questionnaire

Figure 1 provides a CONSORT flow diagram of participants through the study [38] and for the corresponding checklist [39], see Additional file 1. Of the estimated 4000 adolescents who were approached in the schools, about one-quarter were considered eligible based on previous prevalence rates of persistent pain in Western countries [3–7]. The total sample size was the result of three months recruitment in five government-funded high schools. A total of 112 adolescents agreed by email to participate and stated they were in pain, however only 73 participants completed the measures at T0 and were randomized into either control or intervention group. A total of 28 participants (38%) completed the postintervention questionnaire (T1); 17 (23%) were in the intervention group and 11 (15%) the control group, which produced an attrition rate of 62%.

Next, the adolescents’ interactions with the app were analyzed to explore their level of engagement. The daily check-ins for symptom tracking (I) in the app were used by both groups and were categorized as low engagement. Participants in the control group (*n* = 29) conducted a median of 9 (range 1–56) check-ins for symptom tracking (I) during the study period distributed as follows: low (80%), low-moderate (10%), high-moderate (7%) and high (3%). The intervention group (*n* = 31) used all features (I–IV) and conducted a median of 6 (range 2–52) check-ins for symptom tracking (I) distributed as follows: low (74%), low-moderate (13%), high-moderate (10%) and high (3%). The goalsetting feature (II) was used most frequently to set up physical activity goals (43%), such as walking or running, followed by goals related to mood (21%), energy (13%), sleep (13%), and social activities (10%). A median of 3 goals (range 1–156) were set up by the participants (intervention group). The toolbox of pain education and self-management strategies (III) comprised 91 articles, and 8 participants favored 24 of these articles. Articles about coping with fatigue and yoga were most frequently favored, followed by distraction techniques, healthy eating, strength training, and tips for developing a treatment plan. The social support feature (IV) was rarely used. No technical issues were registered.

### Preliminary effectiveness

The demographic and the outcomes variables did not differ significantly between the participants who completed the study (*n* = 33, pain intensity 5.4, SD 1.9) and those who later dropped out (*n* = 40, pain intensity 5.7, SD 2.0).

As anticipated, the scores for all 10 subscales of HRQOL were higher at T1 than at T0 in the intervention group (Table 2). Baseline-adjusted ITT analyses were used to identify possible group differences on pain intensity and HRQOL subscales but showed no significant differences (all *p* > .05).

**Table 2:** Unadjusted descriptive statistics for pain and HRQOL for both groups

Outcomes	Control group Mean [SD]	Intervention group Mean [SD]	Effect size (Cohen’s *d*)
*Pain intensity*			
Before intervention T0	5.5 [1.9]	5.4 [1.9]	.08
After intervention T1^a^	5.1 [1.65]	5.3 [2.45]	
HRQOL			
*Physical well-being*			
T0	50.0 [19.43]	41.6 [21.55]	.16
T1^b^	48.9 [17.56]	52.4 [25.56]	
*Psychological well-being*			
T0	54.5 [20.4]	56.8 [23.1]	.10
T1^b^	63.9 [23.71]	65.9 [20.74]	
*Mood*			
T0	61.6 [18.81]	55.4 [22.72]	.07
T1^b^	68.4 [16.98]	69.7 [20.05]	
*Self-perception*			
T0	46.1 [22.64]	45.7 [23.04]	.01
T1^b^	56.8 [24.46]	56.5 [23.37]	
*Autonomy*			
T0	61.6 [17.80]	56.6 [20.23]	.14
T1^b^	65.7 [15.67]	62.9 [24.05]	
*Parents’ relationship*			
T0	61.2 [23.44]	68.1 [24.64]	.23
T1^b^	65.8 [28.92]	72.1 [26.18]	
*Social support*			
T0	56.6 [23.69]	63.3 [18.40]	.13
T1^b^	66.7 [17.75]	69.1 [20.68]	
*School environment*			
T0	52.7 [20.32]	55.0 [19.77]	.49
T1^b^	57.4 [28.50]	69.1 [16.74]	

^a^Control group *n* = 14, intervention group *n* = 19

^b^Control group *n* = 14, intervention group *n* = 17

No significant group effects were found for the T0-adjusted ITT analyses of self-efficacy, pain self-efficacy, social support, and HADS (all *p* > .05). As shown in Table 3, HADS had a medium effect size (*d* = .53) and HADS-D had a large effect size (*d* = .91).

**Table 3:** Unadjusted descriptive statistics on self-efficacy, pain self-efficacy, social support, and HADS scores for both groups

Secondary outcomes	Control group Mean [SD]	Intervention group Mean [SD]	Effect size (Cohen’s *d*)
*Self-efficacy*			
Pre-intervention T0	13.9 [3.03]	13.0 [3.42]	.22
Post-intervention T1^a^	14.2 [3.02]	14.9 [3.59]	
*HADS-T*			
T0	15.2 [6.14]	16.7 [6.58]	.53
T1^a^	15.0 [5.93]	11.7 [6.31]	
*HADS-A*			
T0	6.3 [2.97]	6.7 [3.74]	.02
T1^a^	5.4 [3.25]	5.5 [3.91]	
HADS-D			
T0	8.9 [3.86]	10.1 [4.14]	.91
T1^a^	9.6 [3.95]	6.2 [3.49]	
*Perceived social support*			
T0	10.3 [2.80]	10.5 [3.24]	.09
T1^b^	10.2 [3.38]	10.5 [4.35]	
*Pain self-efficacy*			
T0	44.4 [11.56]	41.3 [11.37]	.32
T1^c^	48.3 [8.50]	44.5 [14.12]	

^a^Control group *n* = 13, intervention group *n* = 17

^b^Control group *n* = 12, intervention group *n* = 17

^c^Control group *n* = 11, intervention group *n* = 17

**Fig. 2:**
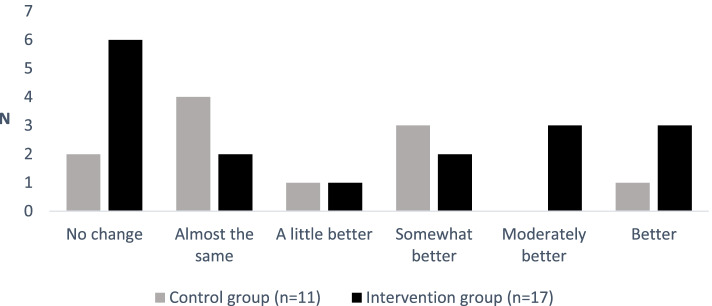
Changes in symptoms since the beginning of the intervention

The self-assessed level of change in symptoms from T0 to T1 showed that about half of the adolescents in both the intervention group (47%) and control group (55%) reported “no change” or “almost the same” since T0 (Fig. 2). One-third of the adolescents in the intervention group (*n* = 6) reported that they felt moderately better or better since T0, whereas only 9% (*n* = 1) in the control group reported this improvement.

## Discussion

This study examined the feasibility of the Norwegian *iCanCope with Pain*^*TM*^ app in an 8-week pilot feasibility RCT among adolescents with persistent pain in a school-based population. We found high attrition rates and a low level of engagement with symptom check-ins, which indicate the need for change in trial design in a future full-scale RCT to improve participant retention.

Despite being underpinned by rigorous adaptation and usability testing [22], this school-based study sample had unexpectedly high attrition rates (62%). High treatment attrition is not uncommon for remotely delivered internet pain management interventions for adolescents with persistent pain [40]. Attrition and loss to follow-up are more common for online trials than for conventional trials, probably because of fewer interactions or less support than in traditional face-to-face trials [41]. For instance, the use of both postal and telephone reminders for follow-up seems to be more effective than texting reminders for follow-up [41]. Given we only used email as a corresponding electronic platform, might have influenced to the lack of response. Another possible explanation for the high attrition rates in our study may be that the app changed the pain experience, as shown by worsening or improving symptoms. Thus, the cyclic nature of persistent pain in adolescence may help explain why some dropped out when feeling better or other to discontinue when feeling worse. Some participants might have perceived the app as a nonpreferred coping method. However, the high attrition rate was not due to technical issues as no technical requests were reported during the intervention period.

Further, the lack of support may be important for several reasons. Support by health-care personnel was one suggestion for improving the usability of the Norwegian *iCanCope with Pain*^TM^ [22]. Studies have shown that adolescents in pain do not know where to seek coping information, and many rely on adults such as their parents [14, 15]. In a meta-analysis, Spek and colleagues found that Internet-based therapy with therapist support is more effective than Internet-based therapy without any support [42]. These findings are in line with a recent review that examined the best evidence for rehabilitation of persistent pain among children and adolescents, which found that multidisciplinary interventions that include an intensive interdisciplinary pain management approach are important [43].

In our study, low engagement was revealed by the adolescents as the few check-ins of symptom tracking (I) in both groups. These findings contrast with those in a recent study by Lalloo et al. [36], who assessed a clinical sample of adolescents in pain recruited from tertiary care centers and reported that the intervention and control groups conducted on average 33.8 and 36 registrations, respectively, and that both were categorized as high-moderate engagement. Differences in engagement between our study and that by Lalloo et al. may relate to difference in samples. For example, our study sample was a school-based population of adolescents with persistent pain, who did not receive support and did not have a clear diagnosis. By contrast, the study sample of Lalloo et al. comprised adolescents recruited from pain clinics and tertiary care centers in ongoing treatment courses who received support and had regular interactions with an interdisciplinary health-care team. Although the frequency of check-in registrations was found similar in the 2 groups in our study, only 1 participant in the control group reported that he or she felt moderately better or better since the beginning of the intervention compared with 6 participants in the intervention group. This finding suggests that other components in the app (II–IV) may be important for pain management.

The ITT analyses (*n* = 19 + 14) revealed no significant differences in outcomes between groups. Still, an important finding was the medium effect size for the HADS total score and large effect size for the HAD-D score. Given that the most liked articles focused on coping with fatigue and distraction techniques*,* these findings suggest that these adolescents with persistent pain found the app to be a relevant tool for learning about cognitive and mental coping strategies. The high mean depression score in this school-based population is in line with previous work that indicated that school-based non-referred adolescents with recurrent headache reported higher depressive symptoms compared with clinically referred adolescents with recurrent headache [44].

Our study has strength and limitations. We found an unexpected high attrition rate that naturally influenced the risk for bias. We, therefore, compared outcomes between participants who completed the study and those who later dropped out. Despite promising usability testing of the Norwegian culturally adapted version, we considered the need for more useful information about the feasibility before running a main RCT. Thus, our initial aim and timeline of a full scale RCT was adjusted, which should be considered as a major limitation as it required retrospectively changes in our trial protocol. Still, as feasibility testing is recommended for reducing uncertainty and increasing the chance of successfully conducting the main RCT [45], we found the pilot feasibility randomized controlled trial to be the most appropriate design and thus provided a solid rationale for the way forward. Another limitation of this study is the onboarding process relying on a PowerPoint presentation, as a total of 13 participants remained unresponsive after randomization. This might indicate the need for a more age-specific onboarding, as for instance the use of digital platforms appropriate for adolescents. Further, given the cyclic nature of pain, it should be considered as a limitation that LPQ only examines pain “at this present moment” and not daily or weekly “average” scores of pain intensity. This was a pilot feasibility RCT, and the sample size was consistent with Hertzog’s recommendations (10–40 responders per group) for pilot studies [46] to allow for exploration of outcomes and the opportunity to estimate the number needed for future definitive trials. A strength of this pilot study is its RCT design combined with assessment of the feasibility of using a rigorously developed, culturally adapted, and well-tested app, which is both theory and evidence based. Given that this was a feasibility study and considered exploratory, we did not consider that adjustments for multiple testing and providing *p* values on demographics were appropriate. Finally, all participants attended the 8-week intervention during the same period from mid-April to mid-June in 2018. This means that the postintervention measurements coincided with the participants’ examination period, which may have induced a higher stress level. We do not know whether this factor affected the attrition rates or any other outcomes.

## Conclusions

High treatment attrition and low engagement rates indicated need for some changes in trial design in a full-scale RCT to improve participant retention. Possible changes in trial design could be including personal support such as more appropriate onboarding using relevant age-specific platforms and use of additional electronic platforms for communication with participants. Nevertheless, our findings provide estimates for calculation of sample sizes in future app-based intervention trials of school-based adolescents with persistent pain.

## Supplementary Information

**Additional file 1.** CONSORT 2010 checklist of information to include when reporting a pilot or feasibility trial*

## Data Availability

The datasets used and/or analyzed during the current study are available from the corresponding author on reasonable request.

## Abbreviations

GSE: General Perceived Self-Efficacy Scale
HADS: Hospital Anxiety and Depression Scale Questionnaire
HADS-D: HADS depression score
HADS-T: HADS total score
HRQOL: Health-related quality of life
ITT: Intention-to-treat
LPQ: Lübeck Pain-Screening Questionnaire
PGIC: Patients’ Global Impression of Change Scale
PSEQ: Pain Self-Efficacy Questionnaire
PSS-Fr: Perceived Social Support–Friends Scale
RCT: Randomized controlled trial
SD: Standard deviation
VAS: Visual analog scale
